# Tuning Surface-Enhanced Raman Scattering (SERS) via Filling Fraction and Period in Gold-Coated Bullseye Gratings

**DOI:** 10.3390/nano15241863

**Published:** 2025-12-11

**Authors:** Ziqi Li, Yaming Cheng, Carlos Fernandes, Xiaolu Wang, Harry E. Ruda

**Affiliations:** Department of Materials Science and Engineering, University of Toronto, Toronto, ON M5S 1A1, Canada; grace.cheng@mail.utoronto.ca (Y.C.); carlos.fernandes@utoronto.ca (C.F.); hazelxiaolu.wang@mail.utoronto.ca (X.W.); harry.ruda@utoronto.ca (H.E.R.)

**Keywords:** surface-enhanced raman scattering (SERS), bullseye plasmonic nano-gratings, localized surface plasmons (LSPs), surface plasmon polaritons (SPPs), electromagnetic field enhancement, plasmonic substrate optimization

## Abstract

Surface-enhanced Raman scattering (SERS) is a highly sensitive analytical technique capable of single-molecule detection, yet its performance strongly depends on the underlying plasmonic architecture. In this study, we developed a robust SERS platform based on long-range–ordered bullseye plasmonic nano-gratings with tunable period and filling fraction, fabricated via electron beam lithography and reactive ion etching and uniformly coated with a thin gold film. These concentric nanostructures support efficient surface plasmon resonance and radial SPP focusing, enabling intense electromagnetic field enhancement across the substrate. Using this platform, we achieved quantitative detection of Rhodamine 6G with enhancement factors of $10^{5}$. Notably, our results reveal a previously unrecognized mechanistic insight: the geometric configuration producing the strongest local electric fields does not yield the highest SERS enhancement, due to misalignment between the dominant field orientation and the molecular polarizability tensor. This finding explains the non-monotonic dependence of SERS performance on grating geometry and introduces a new design principle in which both field strength and field–molecule alignment must be co-optimized. Overall, this work provides a mechanistic framework for rationally engineering plasmonic substrates for sensitive and quantitative molecular detection.

## 1. Introduction

Raman spectroscopy is a powerful, non-destructive, and highly sensitive analytical technique capable of providing molecular fingerprinting as well as structural, chemical, mechanical, and thermal information about a sample [1]. However, its widespread application is limited by the inherently weak signal resulting from the inelastic nature of Raman scattering [2,3]. To overcome this limitation, surface-enhanced Raman spectroscopy (SERS) has been developed, leveraging surface plasmon polaritons (SPPs) to confine, enhance, and localize optical fields at subwavelength scales [4]. Direct excitation of SPPs on smooth metal surfaces is hindered by the wavevector mismatch between incident light and surface modes [5]. Therefore, coupling mechanisms such as prisms, waveguides, optical fibers, or gratings are required to facilitate SPP excitation [6]. Prism-based approaches are more commonly used [7,8,9,10], but the bulky nature of prism-based systems limits their integration into compact or portable sensing devices. By contrast, gratings are particularly attractive due to their high coupling efficiency, compatibility with normal incidence, and tunable optical response through the precise control of geometric (period, height, width) and material parameters [11,12,13].

Compared with conventional one-dimensional gratings or randomly distributed nanopatterns, bullseye gratings offer several unique advantages for SERS. First, their concentric geometry enables radial focusing of SPPs toward a single, well-defined central aperture, creating a highly localized and reproducible hot spot without requiring precise alignment of the excitation beam [14]. Second, the repeating groove–gap structure supports sequential SPP launching from multiple rings, allowing short-range high-intensity near-field enhancements to be repeated and coherently funneled toward the center [15]. This cumulative focusing effect is difficult to achieve in linear gratings, where SPPs propagate along a single direction, and interference is more sensitive to the illumination angle. Third, the rotational symmetry of the bullseye design provides polarization-insensitive coupling and maintains enhancement over a wide range of incident beam orientations [16].

Recent advances in nanophotonics have produced a broad range of nanostructured platforms and design strategies aimed at further improving SERS performance. Nanoparticle-on-mirror (NPoM) and nanoparticle-on-metasurface architectures create ultrasmall metallic nanocavities that support extreme field confinement and have enabled highly sensitive and reproducible SERS detection in both rigid and flexible formats [17,18,19]. Plasmonic nanogap grids and engineered gap antennas—where the gap width, array geometry, and periodicity are controlled with nanometer precision—offer tunable enhancement and have been exploited for strain-sensitive, environment-responsive, and broadband SERS applications [20]. Meanwhile, metasurface-based SERS substrates, including pyramidally perforated films and 3D plasmonic nanoarchitectures, leverage hybrid localized-propagating modes and multi-resonant coupling to achieve ultrahigh enhancement with improved spatial uniformity, polarization robustness, and device-level stability [21]. Recent comprehensive reviews further highlight the shift toward rationally engineered plasmonic and dielectric metasurfaces, flexible sensing platforms, and nanoantenna-type resonators as promising routes for high-performance, application-ready SERS technologies [22,23]. Within this growing landscape, bullseye gratings represent a complementary class of substrates in which SPP focusing, symmetry-enabled mode engineering, and geometric tunability can be systematically correlated with period and filling fraction—yet their SERS-specific behavior remains comparatively underexplored.

Previous studies have investigated periodic rectangular and sinusoidal gratings for SPP-assisted Raman enhancement [24,25,26,27]. However, research on circular grating—where the grooves form concentric rings resembling a “bullseye” pattern-remains limited. In particular, the influence of the grating period (defined as the sum of groove width and spacing) and the filling fraction (the ratio of groove width to period) on the localized electric field and the resulting Raman enhancement factor (EF) has not been thoroughly explored.

The literature suggests that the bullseye geometry holds great potential for SERS, as it has already demonstrated efficient SPP generation in other application fields [28,29,30]. The pioneering work by Lezec et al. [28] demonstrated substantial transmission enhancement through a subwavelength aperture using a bullseye geometry, attributed to the efficient excitation of surface plasmon resonances [31,32,33]. Since then, plasmonic bullseye antennas have been successfully employed in enhanced transmission, energy concentration, beam steering, and polarimetry [34,35,36,37], with analogous microwave leaky-wave antenna designs achieving similar functionalities for high-gain low-profile communications systems [38,39,40].

In the present work, we carried out a careful SERS investigation on a gold-coated bullseye grating. We examined the impact of the array geometry, especially the change in period and filling fraction, on the SERS EF. For this purpose, fifteen unique structures with variations in period and filling fraction were fabricated using the combination of electron beam lithography (EBL) and reactive ion etching (RIE), followed by metallization with gold. Dependences between the period, filling fraction, and SERS enhancement factor were studied from theoretical and experimental points of view. High sensitivity and quantitative detection ability are simultaneously obtained in the bullseye SERS substrates. A low SERS detection limit of $10^{-6}$ M for Rhodamine 6G is realized with EF at $10^{5}$. The relative standard deviation is 2.1–2.9%. Even in a broad concentration range from $10^{-3}$ to $10^{-7}$ M, the SERS intensity and concentration relationship can be fitted as a sigmoidal curve with $R^{2}=0.96$.

## 2. Optical Properties and Physical Background

**Figure 1:**
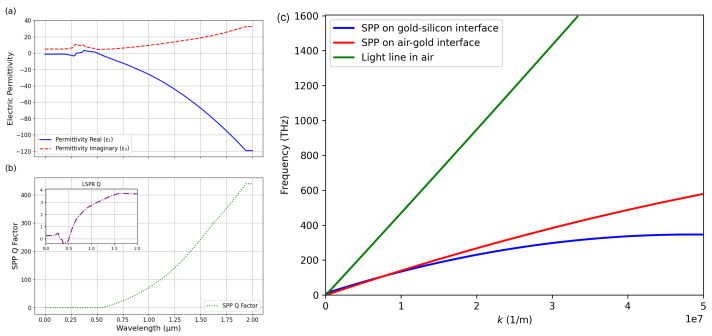
(**a**) Real and imaginary part of the relative permittivity $\epsilon_{m}$ of gold is plotted as a function of wavelength from 0 to 2 μm. (**b**) Localized surface plasmon resonance quality factor and surface plasmon polariton quality factor are plotted for wavelengths from 0 to 2 μm. (**c**) Dispersion curves of light and SPP waves of a gold–silicon interface in air. To allow light to couple to SPPs, the extra momentum is needed to increase the incident light momentum.

To excite an SPP, energy and momentum need to be transferred from a photon or light to the free electron at the interface, resulting in collective oscillations; thus, the momentum must be conserved. The momentum is highly dependent on the dielectric constant (permittivity) of both metal and dielectric materials. In this study, the system is built by a semi-infinite air above a gold–silicon interface (the thickness of the gold is 40 nm). The optical properties of this interface were calculated, and the results are shown in Figure 1a. The permittivity was calculated based on the refractive index (n) and extinction coefficient (k) of each material; the corresponding literature values for the near-infrared region are taken from Johnson and Christy’s data [41]. The permittivity, represented as a complex number $\epsilon =\epsilon^{\prime }-j\epsilon^{\prime \prime }$, indicates the interface’s response to electric field, determining both the confinement and the loss characteristics of surface plasmon modes. A high negative real part of the permittivity indicates that the material supports collective electron oscillations that lag behind the driving field due to electron inertia—an essential condition for plasmonic resonances to happen. The imaginary part of the permittivity quantifies how lossy a material is, representing energy absorption due to internal damping. The larger values indicate higher absorption losses and, therefore, shorter-lived plasmonic excitations.

For better quantification, two types of quality factors (Q factors) can be calculated: they are the surface plasmon polariton ($Q_{SPP}=\frac{Re{\left(\epsilon \right)}^{2}}{Im(\epsilon )}$) and the localized surface plasmon resonance ($Q_{LSPR}=\frac{Re(\epsilon )}{Im(\epsilon )}$), both illustrated in Figure 1b. These expressions capture how the balance between confinement (driven by real part of the permittivity) and loss (driven by imaginary part) determines the plasmonic response. A high $Q_{LSPR}$ corresponds to a sharper and more pronounced localized resonance, which enhanced the electromagnetic near-field strength at the interface. Meanwhile, $Q_{SPP}$ governs the propagation characteristics of SPPs along the planar interface. Because it scales with $Re{\left(\epsilon_{m}\right)}^{2}$, even small reductions in loss can significantly extend the SPP propagation length. The corresponding equations are valid for interfaces of metal and air [4].

Although a high value of $Q_{LSPR}$ correlates with a strong observable plasmon resonance, $Q_{SPP}$ can be used to calculate the propagation length for SPPs at a given wavelength. The propagation length is given by

(1) $$L_{SPP}=\frac{c}{\omega }{\left(\frac{Re\left(\epsilon_{m}\right)+\epsilon_{d}}{Re\left(\epsilon_{m}\right)\cdot \epsilon_{d}}\right)}^{\frac{3}{2}}\frac{Re{\left(\epsilon_{m}\right)}^{2}}{Im(\epsilon_{m})},$$

where *c* is the incident light speed in a vacuum, and ω is the angular frequency of the photon or incident light [42]. Figure 1b shows large values for $Q_{SPP}$, which has low loss and a longer propagation length (∼43 μm, as shown in Figure S1); therefore, gold can be considered a promising candidate for observing SPPs.

The SPP propagation constant at a planar metal–dielectric interface is always larger than light for any given frequency. This wave vector mismatch is represented in Figure 1c, where k is the propagation constant, and the frequency is the angular frequency (ω). The dispersion curves of the SPP mode in air–gold and in the gold–silicon interface are plotted. The intersection between the core mode and the SPP mode dispersion curves determines the phase matching condition. There is no intersection; thus, an additional momentum needs to be added to the momentum of the incident optical wave to match that of the SPPs. The coupling of light to SPPs can respond to light scattering at periodic patterned surfaces, which provides the incident wave with an additional momentum.

**Figure 2:**
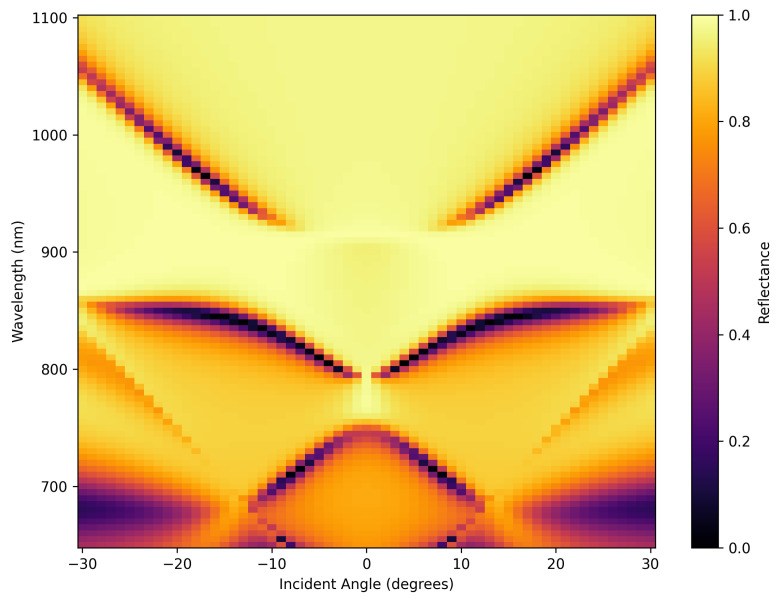
Surface plot depicting the simulated reflectance of a gold grating as a function of wavelength and incident angle.

A grating at this gold–silicon interface is required to couple light from a laser pulse to the SPP. The surface plot in Figure 2 depicts the simulation results of the reflectance spectra calculated for a grating at the gold–silicon interface (width = 100 nm, period = 200 nm, depth = 100 nm) in terms of the incident angle and light wavelength. The simulation was set at various incident angles from −30° to 30° normal to the grating surface and wavelengths from 600 nm to 1100 nm, corresponding to a frequency shift from 2 × $10^{14}$ Hz to 5 × $10^{14}$ Hz. Through the calculation, as the light does not pass through and decays rapidly inside the substrate, the transmission is set to zero (Figure S2a); the weak intensity (dark purple) area of the reflected light indicates that for a certain combination of wavelength and incident angle, the reflected light is minimized, which might be caused by the excitation of SPPs at the metal surface.

The SPP dispersion curve of the grating is shown in Figure S2b. Compared to the flat interface, the grating dispersion curve becomes more curved (higher group velocity) as it introduces momentum states as $\frac{2\pi }{\mathrm{period}}$. The group velocity then can be determined from the angular frequency (ω) with respect to its propagating constant ($k\nu_{g}=\frac{d\omega }{dk_{x}}$), or $\omega =\frac{2\pi c}{\lambda }$, where $k_{x}=\frac{2\pi }{\lambda }\sin\left(\theta \right)$; λ is the wavelength, θ is the incident angle, and *c* is the light speed ($3\times 10^{8}$ m/s). We can calculate the group velocity in the range of visible light (shown in Figure S2c,d); the group velocity is about 9.5 × $10^{7}$ m/s, slower than the speed of light. This occurs when the light interacts with the interface, leading to the collective oscillation of surface electrons, with energy transferred to the metal surface and the light slowing down.

## 3. Materials and Methods

### 3.1. Electromagnetic Field Simulations

Plasmonic “bullseye” gratings were modeled and optimized using the finite-difference time-domain (FDTD) method implemented by Lumerical FDTD Solutions. This method has been widely used to solve Maxwell’s equation in complex geometries, which can simulate the electromagnetic field on plasmonic metal surfaces. FDTD is a time-domain technique with E(t) and H(t), where H is the magnetic field, and E is the electric field. The details of the simulation setup are described as follows:

1. Define the physical structure: A model is created for each fabricated structure based on the dimensions obtained from AFM and SEM image analysis. In this simulation setup, the bullseye grating (in air) was considered as four concentric annular grooves milled into a silicon substrate for 150 nm with a central hole at $r_{c}$ = 50 nm coated with continuous gold (upper and bottom coated 40 nm and sidewall 10 nm). The normalized period is chosen from 0.33 to 1.53 calculated by $\Lambda =\frac{\delta }{\lambda /2n_{eff}}$, where Λ is the normalized period, δ is the physical period defined as the sum of groove width and the distance from the nearest groove, λ is the incident light wavelength, and $n_{eff}$ is the effective refractive index. Different filling fractions (or duty cycles) from 0.2 to 0.8 were considered, which are defined as f = width/Λ.
2. Define a simulation region and boundary conditions: Set a reasonable mesh size; mesh refinement around the rings and cavities are used with a mesh size of 0.04 nm. Adjust the mesh size first in the reasonable direction or geometry. Then, periodic boundary conditions (PBCs) are considered along both the x-direction and y-direction, and perfectly matched layer (PML) boundary conditions with a steep angle profile are selected along the z-direction. Ideally, PML boundaries can absorb all incident light without creating any back reflection.
3. Define a source of light: The incident light, a Gaussian beam of wavelength, propagates along the z-axis to excite the system with an amplitude of 1 and a phase of 0. To re-scale the electric field strength in V/m, E was calculated by $E=\sqrt{\frac{2I}{c\epsilon_{0}}}$, where c is the light speed, $\epsilon_{0}$ is the permittivity of free space, I is P/A, the power of input is 1 W, and the area of the laser focus is 121 μm^2^.
4. Define monitors to record data for analysis: The analysis was performed by considering the “Palik” dispersion curve for silicon [43]), whereas the refractive index of titanium and gold is taken from the CRC handbook [44].

The same 3D modeling task was also performed using Finite Element Analysis (FEM) with COMSOL Multiphysics 6.3. As a direction solution in the frequency domain instead of the time domain, it can also obtain a similar solution by exploiting the Fourier transforms. The model was built using electromagnetic waves and the frequency domain, and the geometrical parameters were set the same as FDTD. The background field refractive index (n) was set as 1 (normal background), the wave intensity (k) was set as $\frac{2\pi }{\lambda n}$, the physical field was a scattering field, and the incident field E_0_ was set as 1 V/m, with the z-axis incidence and the x-direction polarization. The periodic boundary was set to the unit cell in the x and y directions to simulate an infinite repeating pattern in both directions. For the z direction, PMLs were added top and bottom.

Simulations were conducted on a single computer with an Intel i7-12700 CPU with 12 cores and 128 GB RAM. Depending on the geometry, the simulations took from 30 min to 20 h in real time to reach converged solutions.

### 3.2. Fabrication

Fabrication of designed bullseye gratings with different filling fractions was performed using the combination of electron-beam lithography (EBL) and reactive ion etch (RIE), as shown in Figure S6. The silicon was sonicated in 2-propanol (IPA) for 5 min, followed by rinsing in acetone and deionized (DI) water for 1 min. The silicon was then dried with nitrogen gas, baked at 180 °C for 5 min to remove water, and subsequently cooled for 2 min. A single photo resist layer of poly-methyl-methacrylate (PMMA, A3, 950 K 3% dissolved in anisole, supplied by MicroChem Corp. (Westborough, MA, USA)) was spin-coated at a rate of 1000 rpm for 60 s to obtain a 300 nm film, and it was subsequently baked at 180 °C for 60 s to remove any residual solvent.

The silicon sample was loaded on a modified transmission electron microscope (TEM) modified Raith EBPG 5000+ Electron Beam Lithography System (RAITH, Dortmund, Germany) at 100 keV and 10 nA beam current with exposure at 400 μC/cm^2^, which was determined by a dose test. After the resistance-development in isopropyl alcohol (MIBK:IPA) (1:3), the sample was then exposed to SF_6_ plasma at a RF power of 50 W, gas flow rate of 50 sccm, and a chamber pressure of 30 Torr using Oxford Instruments PlasmaPro Estrelas100 DRIE System (Oxford Instruments, Abingdon, Oxfordshire, UK). Finally, a combination of 10 nm titanium (Ti) and 40 nm gold (Au) was deposited onto the sample in an E-beam evaporator (Angstrom Nexdep Electron Beam Evaporator, Angstrom Engineering, Ottawa, ON, Canada) at the deposition rate of 0.5 Å/s for Ti and 0.2 Å/s for Au.

To realize the different geometric dimensions used in this study—including variations in the pitch, groove width, and filling fraction—we generated separate design templates in LayoutEditor, where each structure’s dimensions were explicitly specified in the writing instructions. These layout files were then transferred to the Raith EBPG system (BEAMER 6.1 by GenISys GmbH, Unterhaching, Germany) for proximity-effect correction (PEC) and dose optimization. After PEC, the corrected patterns were written as individual small rectangular write-fields, each containing a single bullseye grating with its prescribed dimensions. This workflow ensured the accurate transfer of the designed pitch and width parameters into the final fabricated structures.

To ensure the high rotational symmetry of the concentric bullseye grooves, several lithographic and processing considerations were implemented. First, the EBL pattern layout was defined using a radially symmetric design grid, and proximity-effect correction was applied to compensate for variations caused by forward scattering, backscattering, and secondary-electron exposure, all of which can otherwise lead to azimuth-dependent broadening of the resistance profile. The use of a high acceleration voltage (100 keV) and a thin PMMA layer (300 nm) minimized beam spreading and reduced long-range exposure contributions from backscattered electrons, which is particularly important for preserving uniform groove and ridge widths around 360°.

Furthermore, during RIE, a low chamber pressure (30 Torr) and stable gas flow ensured uniform etch rates (1000 angstrom/min) across the entire pattern, while careful temperature control (0 °C) prevented local heating that might distort the etch profiles. Collectively, these lithographic and etching measures maintained the radial symmetry of the bullseye structures and ensured consistent critical dimensions for all fabricated filling fractions.

### 3.3. Characterization

The images of the fabricated structure were obtained by scanning electron microscope (SEM) (QUANTA FEG 250, FEI Company, Hillsboro, OR, USA) at high acceleration voltage 5–30 kV, and an energy dispersive X-ray spectroscopy (EDS) detector (JEOL Ltd., Tokyo, Japan) coupled with SEM was used for elemental analysis (mainly gold and silicon). For imaging of the sample before gold coating, the SEM was operated in low vacuum conditions (∼120 Pa residual chamber pressure) to prevent major static charging effects due to the impinging electron beam.

The AFM measurements were obtained with a JPK NanoWizard 4 (JPK Instruments, Berlin, Germany) coupled with an inverted optical fluorescent microscope (Zeiss Axio Observer 7, ZEISS, Oberkochen, Germany) via the Quantitative Imaging modality in air in tapping mode. The cantilever, featuring a shape-nitride lever (SNL)-10 probe (supplied by Bruker Ltd., Billerica, MA, USA) with a spring constant of 0.35 N/m and a 2 nm tip radius, was scanned over the surface at a slower rate.

### 3.4. SERS Measurements

The Raman response of Rhodamine 6G (R6G) on top of the fabricated bullseye grating was recorded using PMCRT site Raman-Renishaw InVia (PMCRT 15-605), which is a commercially available Raman setup with an excitation wavelength at 785 nm. One microliter R6G solution was deposited on top of the fabricated substrate and incubated for 10 min under normal humidity and room temperature. The laser beam was focused on a spot of ∼2 μm in diameter by a microscope objective with a magnification of 20× (NA = 0.4) with 0.18 mW μ$m^{-2}$. All spectra were collected at 60 s and calibrated with respect to the Raman peak of the Si wafer at 520 ${\mathrm{cm}}^{-1}$. For the spot-to-spot Raman measurements, 3–4 points were set and repeated three times in different places with a wavelength of 200 ${\mathrm{cm}}^{-1}$ to 1800 ${\mathrm{cm}}^{-1}$. The collected spectra were further averaged over those positions for each grating. Averaged spectra were baseline corrected, and the lowest signal value was subtracted to remove the baseline, background, cosmic rays, and noise before normalization. The final spectra were normalized using $x=x\Sigma x_{i}$. The intensity of each component (x) is used to quantify the phenotypic diversity. The number of components is i. Spectral data were analyzed using Renishaw WiRE software 3.0 and OriginLab 8.

## 4. Results and Discussion

**Figure 3:**
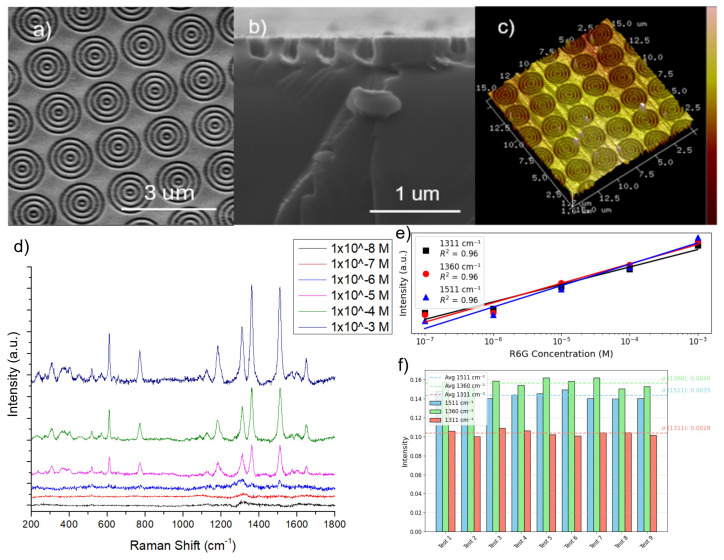
(**a**) Top-view SEM image of bullseye nanostructure arrays; (**b**) side-view cross-section SEM image; (**c**) three-dimensional atomic force microscope (AFM) image. (**d**) Raman spectrum of R6G with different concentrations from $10^{-3}$ M to $10^{-7}$ M from top to bottom; (**e**) quantitative curve between Raman intensity and R6G concentration; (**f**) reproducibility at a concentration of $10^{-3}$ M at 1310, 1360, and 1505 cm^−1^ of the bullseye grating with a filling fraction of 0.5 and a normalized period of 0.509.

Gold-coated bullseye grating arrays were fabricated by a combination of electron beam lithography (EBL) and reactive ion etching (RIE), followed by e-beam gold deposition. The structure has four concentric annular grooves milled into a silicon substrate to a depth of 150 nm using the combination of electron beam lithography and reactive ion etching. Dimensions characterizing the grooves and holes lie in the subwavelength regime (wavelength = 785 nm): all grooves have a depth of 150 nm, and the central hole has a radius of $r_{c}=50\;\mathrm{nm}$. The normalized period is chosen from 0.33 to 1.53 and is calculated by $\Lambda =\delta /(\lambda /\left(2n_{\mathrm{eff}}\right))$, where Λ is the normalized period, δ is the physical period defined as the sum of the groove width and the spacing to the nearest groove, λ is the incident wavelength, and $n_{\mathrm{eff}}$ is the effective refractive index. The effective permittivity can be written as $\varepsilon_{\mathrm{eff}}=f\;\varepsilon_{\mathrm{metal}}+\left(1-f\right)\;\varepsilon_{\mathrm{dielectric}}$, where *f* is the filling fraction (or duty cycle), set from 0.2 to 0.8 and defined as the groove width divided by the physical period. The normalized period is used to account for different materials through the use of an effective refractive index. The number of annular grooves is set to N=4 on each side, which is sufficient to achieve field enhancement. The entire structure is coated with a gold film of thickness 40 nm (a 10 nm titanium adhesion layer is deposited by e-beam evaporation prior to gold deposition). The surface roughness is controlled by tuning the deposition current and rate. The structural unit of the substrate consists of bullseye nanostructures spaced 200 nm apart, forming a long-range ordered array in a rectangular arrangement (Figure 3a–c).

Employing Rhodamine 6G (R6G) as the probe molecule in the range of $10^{-3}$ M to $10^{-7}$ M, we obtained a complete molecular vibration spectrum characterized by distinct and sharp peaks (Figure 3d). R6G is a highly fluorescent rhodamine family dye, which is widely used to determine SERS behavior. The characteristic Raman peaks of R6G at 773 cm^−1^ and 1183 cm^−1^ were assigned to the out-of-plane vibrations and in-plane vibrations of the C-H bond, respectively [45]. The bands at 1310 cm^−1^, 1360 cm^−1^, 1505 cm^−1^, and 1604 cm^−1^ can be attributed to the aromatic C-C stretching vibration modes [45].

It is noted that as the concentration of R6G decreases, the SERS intensity at 1310, 1360, and 1505 cm^−1^ gradually decreases (Figure 3e), which exhibits a linear relationship between the Raman intensity and concentration for all three peaks, with a coefficient of determination (R2) up to 0.96. Additionally, from Figure 3f, ten different random spots were used to acquire Raman spectra of R6G at $10^{-3}$ M, yielding an average RSD of 2.1–2.9% across the three strongest Raman peaks, demonstrating good uniformity. Moreover, each substrate contains a 3×3 array of independently written bullseye patches, and measurements across these physically distinct regions (Figure S5) confirm excellent reproducibility for nominally identical structures.

**Figure 4:**
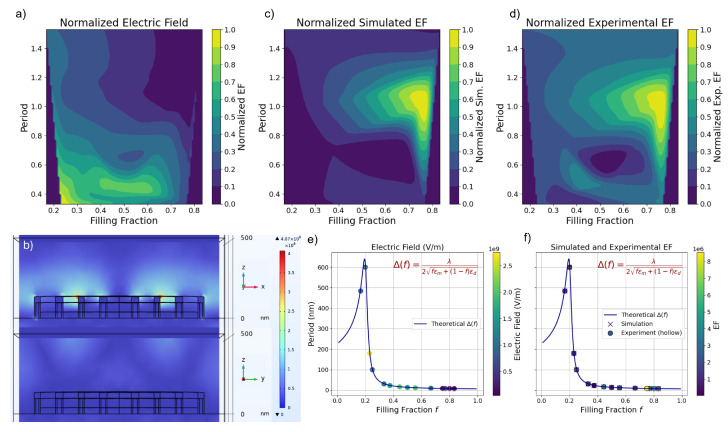
(**a**) Calculated intensity of the local electric field versus the normalized period and filling fraction. (**b**) Three-dimensional electric field distributions (in V/m) in the x-z cross-sectional plane and electric field distributions on the y-z cross-sectional plane of the unit cell gold-coated bullseye grating with a filling fraction of 0.231 and a normalized period of 0.331 under TM illumination. (**c**) Simulated and (**d**) experimentally calculated enhancement factor versus the grating period and filling fraction, where the simulated are superposed with ${\left|E/E_{0}\right|}^{4}$; experimental calculations are determined for the Raman bands at 1360 cm^−1^. (**e**) shows electric field strength, and (**f**) displays simulated EF (marked with crosses) and experimental EF (marked with hollow circles) fitted onto the curve.

From the Raman spectra, we clearly see that the bullseye structure can enhance the Raman signal dramatically. To better understand the physics of plasmonic enhancement and optimize SERS performance, we then modeled electric fields of different geometries using the finite element method (FEM), with Figure 4b showing one geometry as an example. We considered a 500 nm air layer (n = 1) above the gold layer. Since the bullseye is a periodic array, we modeled it by using periodic boundary conditions along the direction of periodicity. The grating was illuminated from the top of the model window at a normal incidence. The illumination was a linearly transverse magnetic (TM) polarized wave. The electric field forms only on the x-z plane due to p-polarization, with no component in the y-direction. Consequently, the strong electric field is concentrated at the sharp groove edges and within the nanogaps as they approach the central aperture.

SPPs are excited through the interaction of the incident light with the gold grating–air interface, generating electric field confinement, as illustrated in Figure 4b. Therefore, the goal is to optimize the SPP resonance matching with a 785 nm adsorption wavelength. The phase matching condition of SPP coupling on a grating is

(2) $$k_{spp}=k_{0}\sin\left(\theta \right)+\frac{2\pi n}{\delta },$$

where $k_{0}$ is the momentum in free space or vacuum, which is $2\pi /\lambda_{0}$; $\lambda_{0}$ is the wavelength of light in vacuum; and *n* is the integer of the diffraction order. In this study, we only discuss *n* = 1 and n=−1, where the brightest diffracted beams (or fringes) are observed, θ is the incidence angle, λ is the 785 nm excitation wavelength, and δ is the grating period.

Periods that satisfy this condition launch SPPs efficiently from each groove, allowing the plasmon waves generated at successive rings to arrive at the central region in phase. This constructive interference yields maximal field buildup at the aperture. Each groove acts as a secondary plasmon source, re-radiating SPP waves toward the center. Adding more grooves increases the number of in-phase contributions and can amplify the field in the central aperture. Because SPPs in gold at 785 nm propagate only tens of micrometers and undergo exponential attenuation, SPPs launched from outer grooves arrive at the center with reduced amplitude and increasing phase dispersion. As shown in Figure S7, the central field rises sharply for the first four grooves and then increases only gradually, indicating that the outer rings provide only a modest incremental contribution to the focusing compared with the dominant coherent contribution from the inner grooves.

To study the period and filling fraction of an isolated dependent electric field at a wavelength of 785 nm, we varied the period and filling fraction of the grating from 0.4 to 1.4 and 0.2 to 0.8, respectively, as shown in Figure 4a. Notice here that the electric field intensity is normalized against the maximum value of 4.45 × $10^{7}$–2.75 × $10^{9}$ V/m to 0–1 to facilitate comparison. The period is also normalized (more details can be found in the experimental information) to minimize the effect of varying the metal-to-air ratio in plasmonic gratings. As a result, at the electric field maximum, reducing the grating period increases the grating momentum $\frac{2\pi n}{\delta }$, which allows phase matching at lower angles. A smaller filling fraction means a narrower groove; the material loss can be reduced, allowing more efficient energy buildup in the plasmonic mode.

From Figure 4c,d, we found that the choice of the grating period and filling fraction can significantly impact the electric field strength; thus, it should also impact the electric field-induced surface-enhanced Raman scattering performance. To compare, one must consider the enhancement factors (EFs). The EFs in experiments were calculated using the commonly used Equation (3):

(3) $$EF=\frac{I_{SERS}/N_{SERS}}{I_{Raman}/N_{Raman}}.$$

where $I_{SERS}$ and $I_{Raman}$ denote the characteristic peak intensities collected over the SERS platform and on the flat gold platform (Figure S3), respectively. $N_{SERS}$ and $N_{Raman}$ are the number of scattering probe molecules on the SERS platform and the flat gold platform in the region of the laser spot, respectively.

It should be noted that the ${EF}_{\mathrm{simulation}}$ is one order-of-magnitude larger than ${EF}_{experiment}$. Ragheb et al. mention that a chemical contribution to the Raman gain may contribute to two orders of magnitude difference [46]. This discrepancy has also been pointed out by Chu et al., who noted similar differences between the simulation and experimental EF values, attributed to fabrication imperfections [47]. After normalization to its maximum, both EFsimulation and EFexperiment show a similar trend, where both maximums correspond to period at 1, given Equation (4). Note also that the very high enhancement at high filling fractions is due to the very high energy density confined to the gap at high filling fractions [48]. Thus, the phase-matching condition is given [49,50] as

(4) $$\delta_{crit}\left(f\right)=\frac{\lambda }{2\sqrt{f\epsilon_{m}\left(\lambda \right)+\left(1-f\right)\epsilon_{d}\left(\lambda \right)}}.$$

Equation (4) shows the critical period ($\delta_{crit}$) versus filling fraction (f), where the light incidence wavelength is 785 nm, $\epsilon_{m}\left(\lambda \right)$ is the gold permittivity, and $\epsilon_{d}\left(\lambda \right)$ is the air permittivity shown in Figure 1a at 785 nm. The dependence of the dielectric constant on the wavelength (λ) arises from the material and waveguide dispersion. We then overlaid data points from previously simulated geometries onto this theoretical curve. These geometries align closely with the prediction, and the color of each point represents the strength of the local electric field or enhancement factor (Figure 4e,f).

When comparing Figure 4e,f, it becomes evident that the bullseye structure exhibiting the highest local electric field strength does not necessarily correspond to the maximum Raman enhancement. In Figure S4, at a filling fraction of 0.75, the lateral plasmonic mode extends across multiple grooves, forming a quasi-continuous channel that guides energy toward the center. This geometry maximizes the effective index matching between adjacent rings and minimizes the phase error accumulated as SPPs propagate inward. Consequently, the SPP waves launched from each groove arrive at the central aperture nearly in phase, producing strong constructive interference and yielding maximal field enhancement. In contrast, at a filling fraction of 0.23, even though the local peak electric field is higher, several grooves behave as isolated scatterers supporting highly localized but weakly coupled gap–plasmon modes. This poor inter-groove coupling prevents efficient energy transfer toward the center and results in weaker overall focusing.

On the other hand, this discrepancy arises because the enhancement is influenced by both electromagnetic (EM) and chemical mechanisms. While EM enhancement—primarily due to field localization—can contribute enhancement factors (EF) exceeding 10^4^, the chemical mechanism (CM), which depends on molecular orientation and polarizability, typically contributes much smaller enhancements (≈$10^{2}$).

(5) $$I_{SERS}\propto {\left|\vec{E_{loc}}\cdot \frac{\partial \vec{\alpha }}{\partial Q}\right|}^{2},$$

where $I_{SERS}$ is the enhancement signal, $\vec{E_{loc}}$ is the enhanced local electric field near the nanostructure due to plasmonic effects, and $\frac{\partial \vec{\alpha }}{\partial Q}$ indicates which direction in space the molecular polarizability changes most when that vibration occurs. If $\vec{E_{loc}}$ and $\frac{\partial \vec{\alpha }}{\partial Q}$ are aligned, their dot product is maximized, and the enhancement is strong; their dot product is maximized, resulting in strong Raman enhancement. If they are orthogonal, the dot product is zero, and the enhancement is suppressed.

To assess this alignment, we examined how the electric field direction—controlled by nanostructure geometry—overlaps with the orientation of the R6G molecule. We found that bullseye structures with higher filling fractions (around 0.8) produce more vertically oriented electric fields (Figure S4), which better match the known vertical orientation of R6G on gold surfaces. Ujihara et al. [51] reported that R6G molecules adsorbed on Au nanoparticles are predominantly oriented perpendicular to the surface [51], and Moretti et al. observed similar upright orientations on nanostructured gold substrates [52]. This vertical alignment enhances the coupling between the transition dipole moment of R6G and the local field, leading to stronger Raman signals. Thus, optimizing SERS performance requires not only maximizing the electric field strength through nanostructure design but also aligning the local field direction with the molecular orientation to fully exploit both electromagnetic and chemical enhancement mechanisms.

## 5. Conclusions

We designed and fabricated periodic gold-coated bullseye gratings on silicon substrates with systematically varied periods and filling fractions. The combination of localized surface plasmon (LSP) excitation in gold and the bullseye grating’s ability to support surface plasmon polaritons (SPPs) resulted in strong electric field enhancement at the substrate surface. The near-field intensity peaked for structures with filling fractions below 0.6 and periods shorter than 100 nm. For SERS evaluation, Rhodamine 6G (R6G) molecules were deposited onto the fabricated substrates. We investigated how the coupling between LSPs and SPPs influences SERS performance. Both experimental and theoretical analyses revealed significant near-field enhancement. Interestingly, we observed that the configuration yielding the strongest electric field did not necessarily correspond to the highest SERS enhancement factor, which we attribute to mismatches in the spatial location and orientation of the enhanced field relative to the analyte molecules. These findings provide valuable guidance for optimizing plasmonic substrates to enhance molecular detection sensitivity in SERS applications.

## Data Availability

The original contributions presented in this study are included in the article/Supplementary Materials. Further inquiries can be directed to the corresponding author.

## Supplementary Materials

The following supporting information can be downloaded at: https://www.mdpi.com/article/10.3390/nano15241863/s1, refs. [53,54,55,56,57,58,59,60,61] are cited in the Supplementary Materials.
