# Supplementary material for: Tuning Surface-Enhanced Raman Scattering (SERS) via Filling Fraction and Period in Gold-Coated Bullseye Gratings
Source: Nanomaterials (Basel). 2025 Dec 11;15(24):1863. doi: 10.3390/nano15241863 (PMC12735832; doi:10.3390/nano15241863)
Supplement: Supplementary file 1 [file nanomaterials-15-01863-s001.zip › nanomaterials-4030544-supplementary.pdf]

Supplementary Materials: Tuning Surface-Enhanced Raman Scattering (SERS) via Filling Fraction and Period in Gold-Coated Bullseye Gratings

Ziqi Li [1], Yaming Cheng [1], Carlos Fernandes [1], Xiaolu Wang [1] and Harry E. Ruda [1]

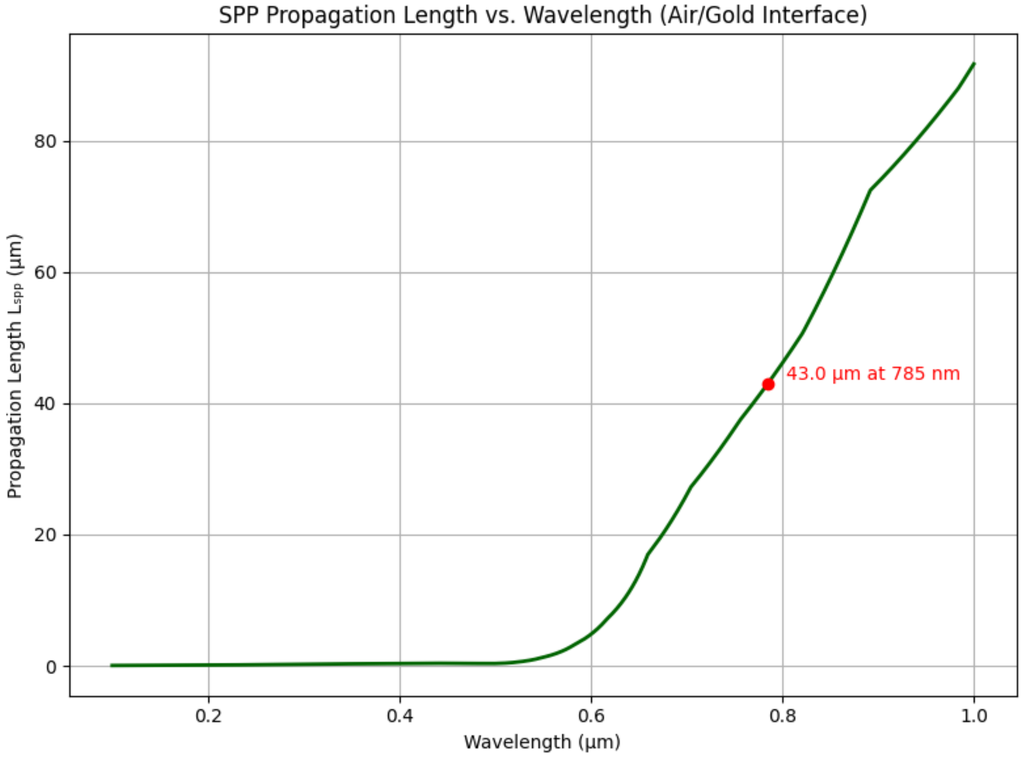

Figure S1. SP propagation length L_{SPP} versus wavelength of gold thin film on silicon 40 nm for excitation wavelengths of 785 nm.

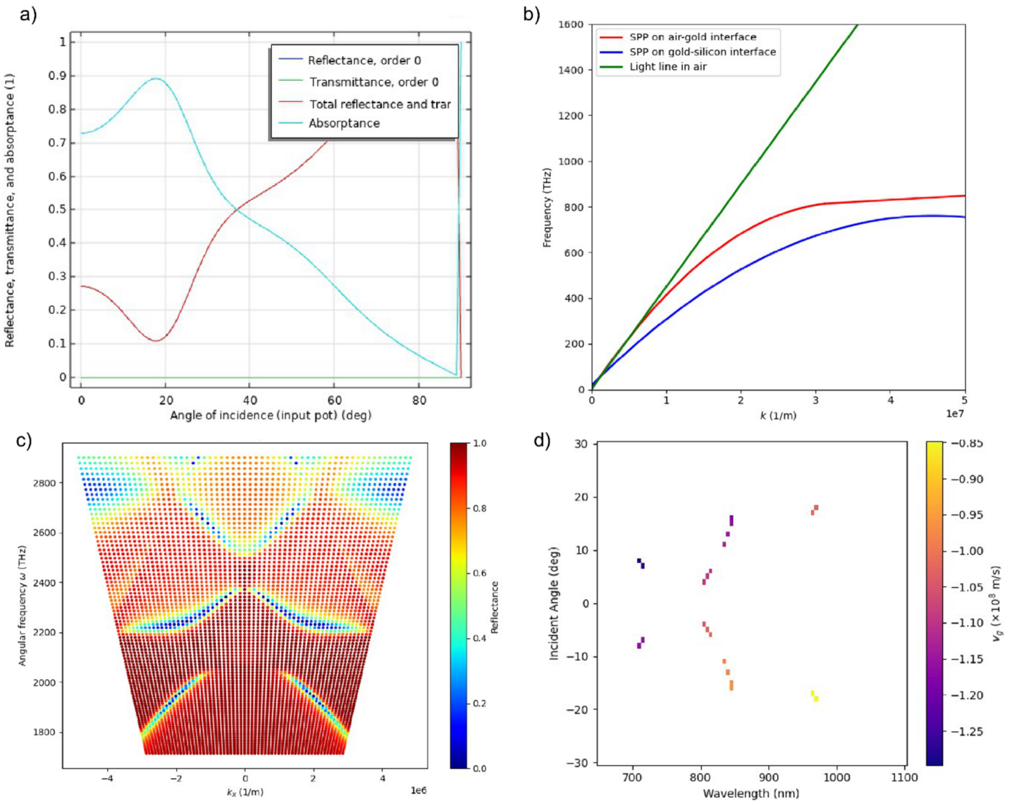

Figure S2. (a) Reflectance, transmittance, and absorptance versus incident angle curve of gold grating at a wavelength of 785 nm. (b) Dispersion curves of the SPP and the incident light of the gold grating, where k is the propagation constant and frequency is the angular frequency. Focusing on the air-gold interface curve (red), for small k corresponding to low (mid-infrared or lower) frequencies, the SPP propagation constant is close to k0 at the light line; for large k, the frequency of the SPPs approaches the characteristic surface plasmon frequency. (c) Surface plot depicting the reflectance of a gold grating as a function of the angular frequency (ω) in THz with respect to its angular wavenumber (kx) in 1/m. (d) Surface plot depicting the group velocity of a gold grating as a function of incident angle and wavelength, indicated by colorful dots.

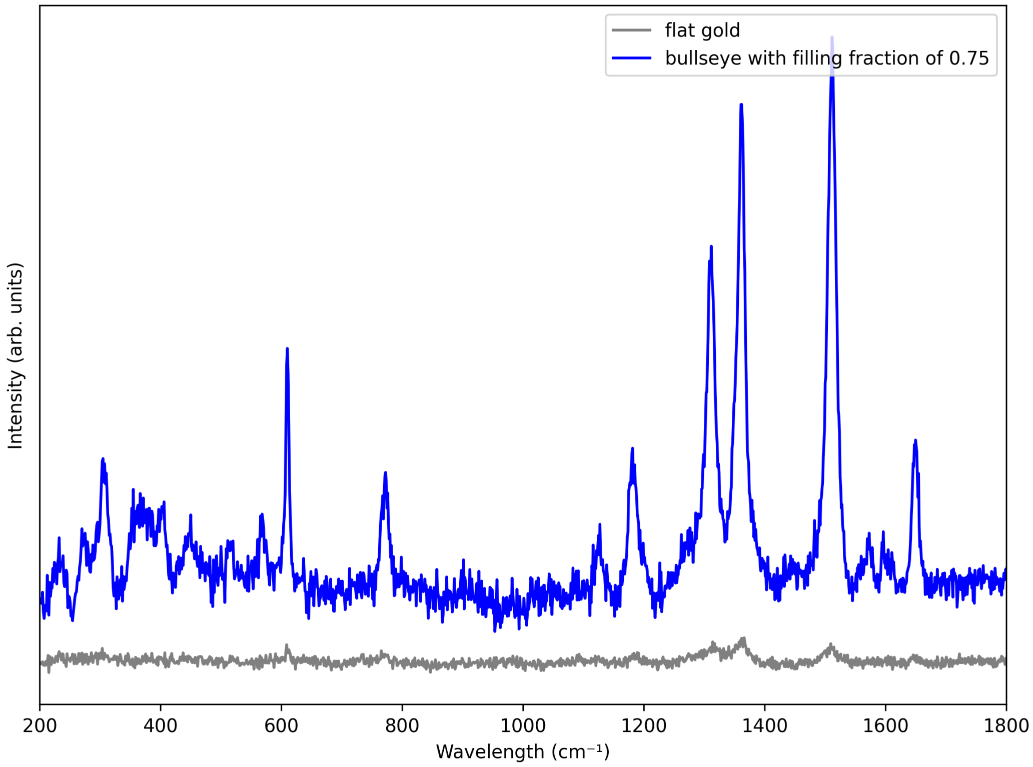

Figure S3. Control experiments showing Raman spectra acquired on bullseye nanogratings compared with those obtained on a planar gold film with R6G at 10^{-3} M . The bullseye structure exhibits stronger and more distinct Raman features, confirming the plasmonic enhancement provided by the grating geometry.

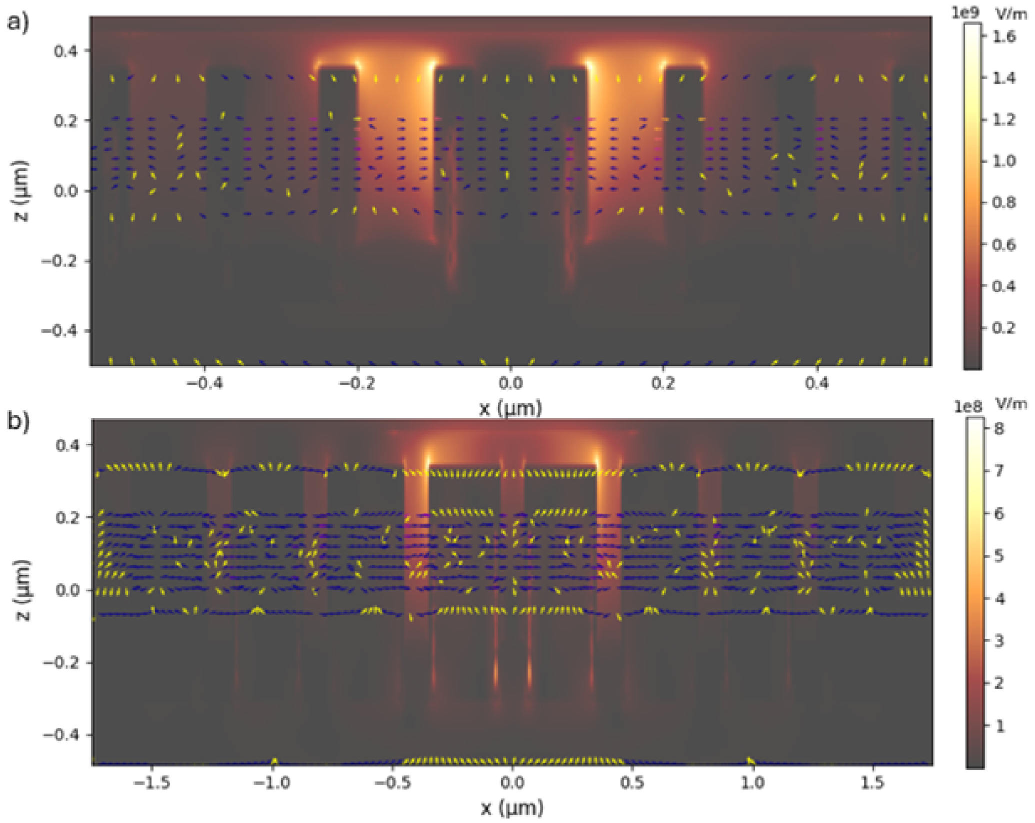

Figure S4. (a) FDTD simulation of electric field intensity in the x-z plane of bullseye with filling fraction of 0.23; (b) FDTD simulation of electric field intensity in x-z plane of bullseye with filling fraction of 0.75. The blue narrows represent the electric vector in horizontal direction and the yellow narrows represent the electric vector in vertical direction ($\left|\frac{E_z}{E_x}\right| > 0.8$). The electric field intensity of the bullseye with a filling fraction of 0.23 is higher, especially near the center of grooves, other the other hand, the bullseye with filling fraction of 0.75 shows lower electric field value, but at the central of electric field vectors, the field vectors are more aligned along the z-axis, indicating a vertical direction.

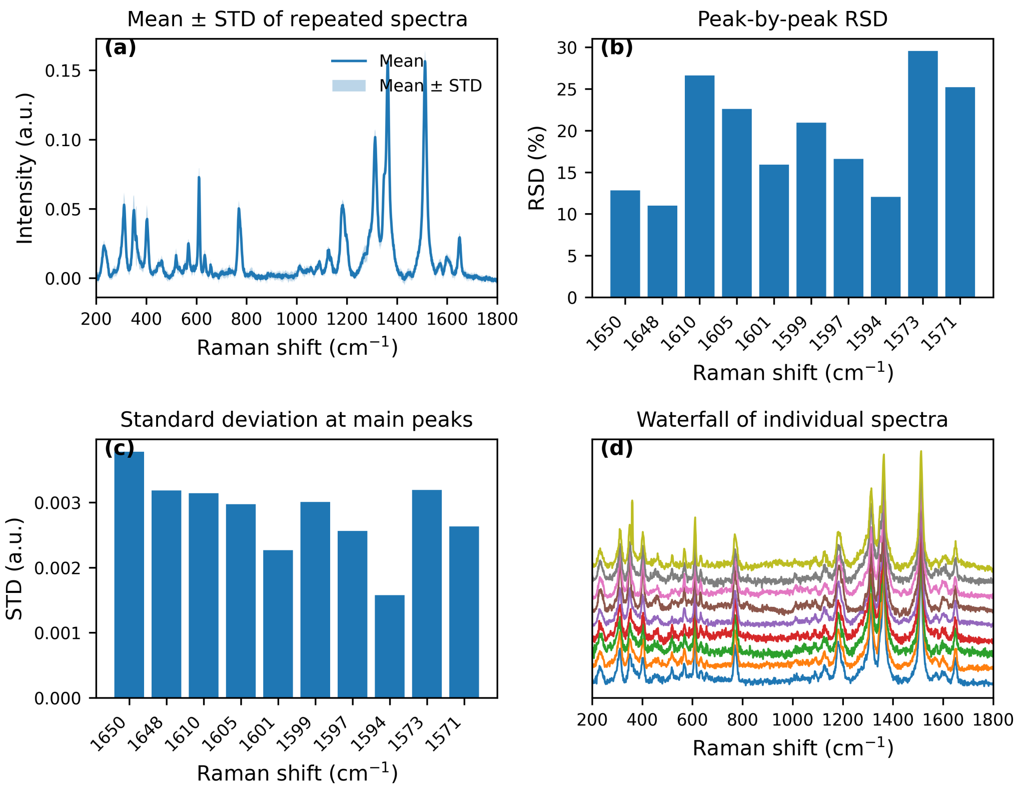

Figure S5. Reproducibility of SERS measurements on the bullseye substrate with filling fraction of 0.75 with R6G concentration of 10^{-3} M. Each bullseye substrate was patterned by electron-beam lithography as a 3×3 array of identical bullseye patches. To assess device-to-device reproducibility for structures with the same nominal geometry, SERS measurements were collected independently from all nine lithographically distinct patches on the substrate. (a) Mean Raman spectrum with shaded standard deviation (STD). (b) Peak-wise relative standard deviation (RSD). (c) Absolute STD at selected Raman modes. (d) Waterfall plot of all repeated spectra.

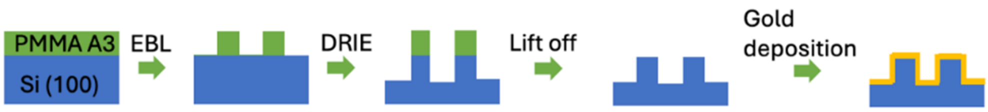

Figure S6. Fabrication scheme. Nanostructure was first patterned on the PMMA A3 layer using EBL. Then, reactive ion etching (RIE) is used to dry etch. The gold was deposited on the surface, and the lift-off process removed the excess.

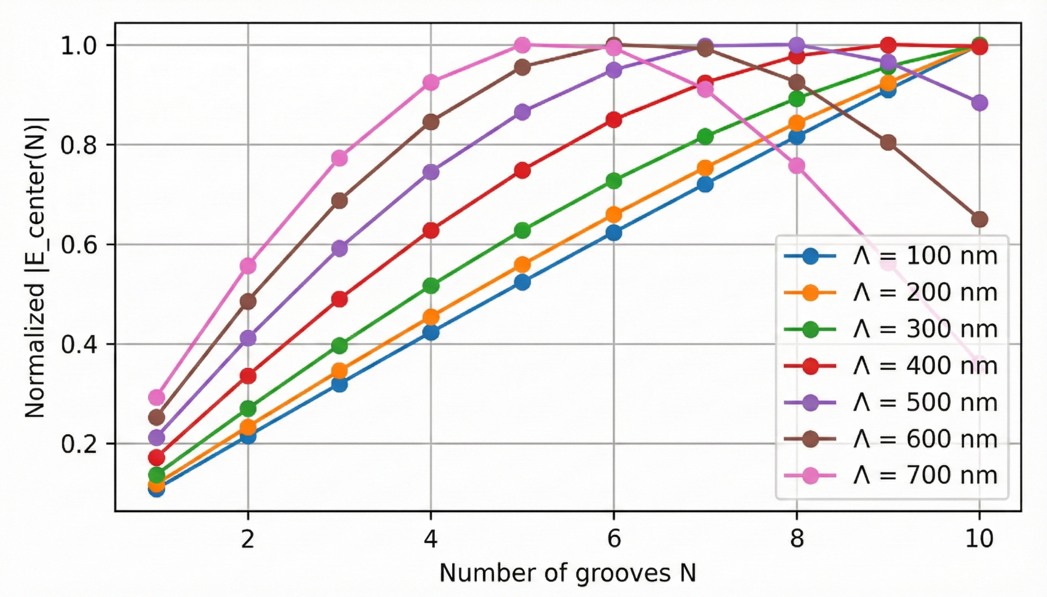

Figure S7. Normalized central field amplitude $|E_{\text{center}}(N)|$, normalized to its maximum value for each curve, as a function of the number of grooves N for several groove periods Λ. For all Λ, the response increases nearly linearly over the first few grooves, reflecting the strong and coherent SPP contributions from the innermost rings that dominate the hotspot formation. As N increases beyond 4–5, the growth rate slows because SPPs launched from outer grooves experience greater propagation loss and increasing phase deviation, reducing their ability to interfere constructively at the centre. The effect becomes more pronounced for larger Λ: when the groove spacing increases, the outer rings are farther from the aperture, so their SPPs accumulate greater loss and phase dispersion, causing the field to saturate earlier and, for the largest Λ values, to reach a maximum around $N \approx 5$ before decreasing.

Table S1. Recent (2020–2025) gold-based SERS substrates and their analytical performance.

Substrate	Analyte	Analytical Parameters (EF, LOD)	Ref
Self-assembled Au nanoparticles (14–99 nm)	Chlorogenic acid	EF $\approx 4 \times 10^4$; uniformity std. $< 15\%$; LOD in μM range	[1]
Au nanostructures via solid-state dewetting	R6G	Moderate enhancement; AEF = $123\times$ (vs. flat Au); RSD $\approx$ 18.5%	[2]
Gold nanoparticles on reduced graphene oxide (Au/rGO)	R6G	EF $\approx 2.7 \times 10^7$; LOD $\sim 10^{-10}$ M	[3]
Au–Ag bimetallic NPs on reduced graphene oxide	R6G	EF $\approx 1.1 \times 10^8$; LOD $\sim 10^{-10}$ M	[3]
High-performance Au NP substrate (optimized synthesis)	R6G	LOD = 10^{-11} M; high EF (as reported in study)	[4]
Au nanohole / metasurface platforms (post-2020 designs)	R6G, MB, 4-MBA	EF 10^6–10^8; LOD often 10^{-9}–10^{-12} M depending on resonance matching	[5]

1. Supporting Text S1 Enhancement Factor (EF) calculation:

SERS enhancement factors (EFs) for all types of bullseye nanostructured substrates were calculated using a commonly used formula,

$$EF = \frac{I_{SERS}}{I_{Raman}} \times \frac{N_{Raman}}{N_{SERS}} \tag{1}$$

where, I_{SERS} is the peak intensity of the SERS substrate. I_{Raman} is the peak intensity of normal Raman, which is Au-coated silicon (reference) in our case. N_{Raman} is the number of probed molecules of the bulk R 6G solution is a normal Raman measurement. I_{Raman} is the normal Raman intensity of the R 6G at the band of 1361 cm-1 in the bulk solution and N_{Raman} is the number of molecules within the reference bulk solution. N_{Raman} was calculated by the following equation:

We used I_{SERS} and I_{Raman} which are the intensity of the band of 1361 cm^{-1}. Based on Kudelski's work, the average area of 1 R6G molecule $\sim$ 8 nm^2 for 10^{-4} M solution based on ref (A. Kudelski, A. Raman, Studies of rhodamine 6G and crystal violet sub-monolayers on electrochemically roughened silver substrates: do dye molecules adsorb preferentially on highly SERS-active sites, Chem. Phys. Lett. 414 (2005) 271–275.)

$$Surface\ density = 1 molecule/(1e^{-6})\mu m^2 = 1.25 \times 10^5 molecule/\mu m^2 \tag{2}$$

With the surface density of R 6G, we then need to calculate the total surface area covered by the laser beam. The surface area can be separated into two parts: the top and bottom flat surfaces and the side wall surface. The total surface area of the top and bottom flat surfaces is the same as the surface area covered by the laser beam (the radius of the laser spot is 11 μm). The area of the side wall is calculated by the following equation,

$$Wall\ surface\ area \times etching\ rate \times etching\ time \times 2 \times \pi \times (\Sigma_{i=1}^{n} r_i + \Sigma_{i=1}^{n} R_i) \tag{3}$$

where r is inner radii of the i-th ring and R is the outer radii of the i-th ring.
surface

$$N_{SERS} \text{ for the top and bottom flat} \tag{4}$$
$$= Surface\ area \times Surface\ density \times concentration\ of\ solution \tag{5}$$
$$= (Same\ area\ for\ Raman) \times density \times c \tag{6}$$
$$= (\pi \times (11 \mu m)^2) \times 1.25 \times 10^5 \frac{molecule}{\mu m^2} \times (1 \times 10^{-4})M \div (5 \times 10^{-4})M \tag{7}$$
$$= 9.49 \times 10^6 \tag{8}$$

$$N_{SERS} \text{ for the side wall surface} \tag{9}$$
$$= Surface\ area \times Surface\ density \times concentration\ of\ solution \tag{10}$$
$$= (Side\ wall\ area) \times density \times c \tag{11}$$
$$= ((150nm \times 2\pi \times (50nm \times 8 + 16 \times 100nm + 12 \times 300nm) \times (11\mu m \div 2 \times (50nm + \tag{12}$$
$$4 \times 100nm + 3 \times 300nm + 100) + \pi \times (11\mu m)^2) \times 1.25 + 10^5 \frac{molecule}{\mu m^2} \times (1 \times 10^{-4})M \div \tag{13}$$
$$= 4.4 \times 10^6 \tag{14}$$

We then need to calculate the N_{Raman} value following the same method, the laser spot size determines the surface area, the concentration for the Raman measurement is 0.1 M (to obtain a clear Raman spectrum), and the surface density is calculated by the Langmuir isotherm model (Figure S8).

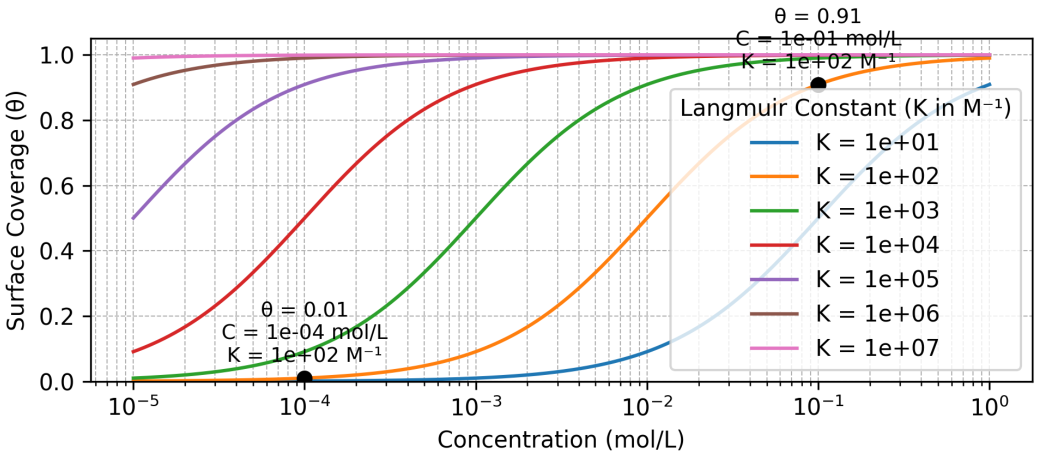

Figure S8. Langmuir adsorption isotherm obtained by plotting the surface coverage (saturation responses at each concentration) versus the corresponding target concentration, plotting based on equation theta = (K * C) / (1 + K * C), where theta is the surface coverage, K is Langmuir constant in M-1 and C is the concentration in mol/L or M.

The Langmuir constant is determined by the following table (Table S2). The organic dye typically exhibits- low to moderate affinity (approximately 0.001 - 0.1 L/mg). In this study, the surface is a continuous gold layer rather than gold nanoparticles, as a flat surface has a lower total active surface area compared to particles. Also, compared to the mesoporous surface (MCM), the continuous gold surface has less affinity as well; thus, the estimated Langmuir constant for R6G on both the flat gold surface and grating gold surface is about 0.001 – 0.01 L/mg or 479 – 4790 M^{-1} using the following equation.

$$KL(M-1) = KL(L/mg)\text{Molar mass of R6G} = \text{KL(L/mg)479000mg/L} \tag{15}$$

Table S2. Langmuir constant for common organic dyes on different materials.

**Adsorbent**	**Adsorbate**	K_L **(L/mg)**	**Reference**
MCM-41	R6G	0.02	[6]
Al-MCM-41	R6G	0.03	[6]
Activated Carbon	R6G	∼0.008–0.02	[7]
TiO_2 nanoparticles	Methylene Blue	∼0.1–0.5	[8]
Au nanoparticles	R6G	∼0.1–0.4 (est.)	[9]

Therefore, considering the maximum surface coverage situation, the surface coverage for 0.1 M on flat gold (0.99) will be one order of magnitude denser than 10^{-4} M on bullseye substrate (0.09).

$$N_{Raman} \tag{16}$$

$$= Surface\ area \times Surface\ density \times concentration\ of\ solution \tag{17}$$

$$= \pi r^2 \times 1.25 \times 10^5 \frac{molecule}{\mu m^2} \times (1 \times 10^{-1})M \div (5 \times 10^{-4})M \div 0.09 \tag{18}$$

$$= \pi \times (11\mu m)^2 \times 1.25 \times 10^5 \frac{molecule}{\mu m^2} \times (1 \times 10^{-1})M \div (5 \times 10^{-4})M \div 0.09 \tag{19}$$

$$= 1.06 \times 10^{11} molecules \tag{20}$$

$$\frac{N_{Raman}}{N_{SERS}} = 10674 \tag{21}$$

$$EF = \frac{I_{SERS}}{I_{Raman}} \times \frac{N_{Raman}}{N_{SERS}} = 10674 \times 10.74 = 1.15 \times 10^5 \tag{22}$$

Supporting Text S2 Semi-analytical SPP Interference Model with Monte Carlo Evaluation of Groove Contributions

To support our claim that only the innermost ~ 4 grooves contribute significantly to the central field enhancement (Figure S7), we implemented a simple semi-analytical SPP interference model for the bullseye structure. We treat each groove as a secondary SPP source located at radius $r_n = n\Lambda$ ($n = 1, \ldots, N$), where Λ is the groove period. The complex field contribution from groove n at the aperture is written as

$$E_n = A_0 \exp\left(-\frac{r_n}{L_{\mathrm{SPP}}}\right) \exp[i(k_{\mathrm{SPP}} r_n + \delta\phi_n)], \tag{23}$$

where L_{SPP} is the SPP propagation length on the Au/air interface at $\lambda = 785$ nm, and k_{SPP} is the complex SPP wavevector,

$$k_{\mathrm{SPP}} = k_0 \sqrt{\frac{\varepsilon_m \varepsilon_d}{\varepsilon_m + \varepsilon_d}}, \quad L_{\mathrm{SPP}} = \frac{1}{2\,\mathrm{Im}\, k_{\mathrm{SPP}}}. \tag{24}$$

Here $k_0 = 2\pi/\lambda$, $\varepsilon_d = 1$ (air), and ε_m is taken from the Johnson–Christy optical constants for gold. The total field at the center from N grooves is

$$E_{\mathrm{center}}(N) = \sum_{n=1}^{N} E_n. \tag{25}$$

To account for fabrication-induced phase errors and surface roughness, we include a stochastic phase term $\delta\phi_n$ whose variance increases with propagation distance,

$$\delta\phi_n \sim \mathcal{N}\left(0, \sigma_n^2\right), \qquad \text{where } \sigma_n^2 = \sigma_0^2 \frac{r_n}{\Lambda}. \tag{26}$$

where σ_0 is the phase standard deviation per groove. We then evaluate $|E_{\mathrm{center}}(N)|$ by Monte Carlo averaging over many realizations of $\{\delta\phi_n\}$.

References

1. Pavlova, A.; Nikolov, A.; Ivanova, T.; et al.. Self-Assembled Gold Nanoparticles as Reusable SERS Substrates Fabricated by the Aqua-Print Method. *International Journal of Molecular Sciences* **2024**, *25*, 12785. https://doi.org/10.3390/ijms252312785.
2. Visbal, M.; Barrera, E.; Raigoza, C.; et al.. The Fabrication of Gold Nanostructures as SERS Substrates for the Detection of Contaminants in Water. *Nanomaterials* **2024**, *14*, 1525. https://doi.org/10.3390/nano14181525.
3. Pillai, P.; Thomas, T.; et al.. Enhancement Factors of SERS Substrates Based on Monometallic and Bimetallic Nanoparticles Embedded on Reduced Graphene Oxide. *Plasmonics* **2025**, *20*, 113–125. https://doi.org/10.1007/s11468-025-03311-x.
4. Yao, X.; Zhao, Y.; Ren, J.; et al.. High-Performance SERS Substrate Based on Gold Nanoparticles. *Materials Today Physics* **2024**, *38*, 101386. https://doi.org/10.1016/j.mtphys.2024.101386.
5. Kim, H.; Park, J.; et al.. Au Nanohole Array Metasurfaces for Multi-Wavelength and Ultra-Sensitive SERS Sensing. *Nano Research* **2022**, *15*, 8290–8303. https://doi.org/10.1007/s12274-021-3827-4.
6. Douba, H.; Mohammedi, O.; Cheknane, B. Adsorption of Rhodamine 6G Dye onto Al MCM-41 and MCM-41 Mesoporous Materials. *Kemija u Industriji* **2022**, *71*, 569–582. https://doi.org/10.15255/KUI.2022.001.
7. Annadurai, G.; Juang, R.S.; Lee, D.J. Adsorption of Rhodamine 6G from Aqueous Solutions on Activated Carbon. *Journal of Environmental Science and Health, Part A* **2001**, *36*, 715–725. https://doi.org/10.1081/ESE-100103755.
8. Du, Y.; Zheng, P. Adsorption and Photodegradation of Methylene Blue on TiO_2 Halloysite Adsorbents. *Korean Journal of Chemical Engineering* **2014**, *31*, 2051–2056. https://doi.org/10.1007/s11814-014-0162-8.
9. Chen, J.; Mårtensson, T.; Dick, K.A.; Deppert, K.; Xu, H.Q.; Samuelson, L.; Xu, H. Surface-Enhanced Raman Scattering of Rhodamine 6G on Nanowire Arrays Decorated with Gold Nanoparticles. *Nanotechnology* **2008**, *19*, 275712. https://doi.org/10.1088/0957-4484/19/27/275712.

Disclaimer/Publisher's Note: The statements, opinions and data contained in all publications are solely those of the individual author(s) and contributor(s) and not of MDPI and/or the editor(s). MDPI and/or the editor(s) disclaim responsibility for any injury to people or property resulting from any ideas, methods, instructions or products referred to in the content.
